# Supplementary material for: Effect of famotidine on hospitalized patients with COVID-19: A systematic review and meta-analysis
Source: PLoS One. 2021 Nov 4;16(11):e0259514. doi: 10.1371/journal.pone.0259514 (PMC8568101; doi:10.1371/journal.pone.0259514)
Supplement: S1 Appendix — (DOCX) [file pone.0259514.s002.docx]

**S1 Appendix.** **Search Strategy**

Database: Ovid MEDLINE(R) ALL <1946 to February 10, 2021>

1      (covid 19 or covid-19).mp. (98012)

2      "coronavirus disease 2019".mp. (18967)

3      SARS-CoV-2.mp. (39939)

4      severe acute respiratory syndrome coronavirus 2.mp. (39862)

5      1 or 2 or 3 or 4 (101636)

6      exp Famotidine/ (1613)

7      "famotidine".mp. (2318)

8      6 or 7 (2318)

9      5 and 8 (33)

Database: Embase <1974 to 2021 February 11>

1      (covid 19 or covid-19).mp. (87363)

2      "coronavirus disease 2019".mp. (87063)

3      SARS-CoV-2.mp. (31402)

4      severe acute respiratory syndrome coronavirus 2.mp. (27219)

5      1 or 2 or 3 or 4 (101544)

6      exp Famotidine/ (9183)

7      "famotidine".mp. (9369)

8      6 or 7 (9369)

9      5 and 8 (66)

Database: EBM Reviews - Cochrane Central Register of Controlled Trials <January 2021>

1      (covid 19 or covid-19).mp. (3882)

2      "coronavirus disease 2019".mp. (1027)

3      SARS-CoV-2.mp. (213)

4      severe acute respiratory syndrome coronavirus 2.mp. (645)

5      1 or 2 or 3 or 4 (3988)

6      exp Famotidine/ (464)

7      "famotidine".mp. (972)

8      6 or 7 (972)

9      5 and 8 (7)

Database: medRxiv <February 12, 2021>

1      (covid 19 OR coronavirus 19) AND famotidine  (3)

Database: researchsquare <February 12, 2021>

1      (covid 19 OR coronavirus 19) AND famotidine  (0)
