# Supplementary material for: Effect of famotidine on hospitalized patients with COVID-19: A systematic review and meta-analysis
Source: PLoS One. 2021 Nov 4;16(11):e0259514. doi: 10.1371/journal.pone.0259514 (PMC8568101; doi:10.1371/journal.pone.0259514)
Supplement: S2 Appendix — (DOCX) [file pone.0259514.s003.docx]

**S2 Appendix.** **PRISMA Flow Diagram**

8 studies identified for potential quantitative synthesis

48 records excluded

28 full-text articles assessed for eligibility

76 records screened after duplicates removed

106 records identified through database search

3 additional records identified

2 studies were excluded because they included non-hospitalized patients

4 studies included in quantitative synthesis (meta-analysis)

2 did not report adjusted relative risk ratios and were excluded

20 full-text articles excluded:

- Commentary article (n = 5)
- Review article (n =4)
- Study protocol (n =1)
- Case study/series (n=2)
- No full text (n=2)
- Non-clinical study (n=6)
